# Supplementary material for: Participation and Adherence to Prehabilitation Programs for Colorectal Cancer
Source: Nutrients. 2025 May 25;17(11):1792. doi: 10.3390/nu17111792 (PMC12157972; doi:10.3390/nu17111792)
Supplement: Supplementary file 1 [file nutrients-17-01792-s001.zip › 20250421PACE_SR_In-_exclusion_Table S1.pdf]

Table S1. Inclusion and exclusion criteria

			Inclusion criteria							Exclusion criteria								
**Author**	**Outcome**	**Patients**	**Age**	**Physical function**	**Nutritional st.**	**Surg.**	**NCRT[10]**	**Tumor**	**Other**	**Physical function**	**Surgery**	**Disease**	**Prehab**	**Postop**	**Treatment**	**Data**	**Other**	**Method recruitment**
Atoui 2023	Other	CRC[1]	≥ 18 yrs.	NC[5]	NC[5]	Elect.	NC[5]	Malignant	NC[5]	Limiting physical function	NC[5]	NC[5]	NC[5]	NC[5]	NC[5]	NC[5]	Other	Outpatient clinic
Berkel 2022	Complication	CRC[1]	Other	VAT[6] /V02 peak/ CPET[7] criteria	Other	Elect.	NC[5]	Benign & malignant	Other	NC[5]	Other	NC[5]	NC[5]	NC[5]	NC[5]	NC[5]	NC[5]	Outpatient clinic
Boereboom 2019	Physical improvement and feasibility	CRC[1]	NC[5]	NC[5]	NC[5]	NC[5]	No	Malignant	NC[5]	NC[5]	NC[5]	NC[5]	Unable to exercise suff.	NC[5]	NC[5]	NC[5]	Unable to provide consent	Outpatient clinic
Bojesen 2022	Feasibility	CRC[1]	NC[5]	WHO[8] < 2	NC[5]	Elect.	No	Malignant	NC[5]	Physical and psychiatric disease	Other	Metastatic disease	Unable to exercise suff.	NC[5]	NC[5]	NC[5]	Other	Outpatient clinic
Bousquet-Dion 2018	Other	CRC[1]	≥ 18 yrs.	NC[5]	NC[5]	NC[5]	NC[5]	Malignant	NC[5]	NC[5]	NC[5]	Metastatic disease	Unable to exercise suff.	NC[5]	NC[5]	NC[5]	Poor language skills	Outpatient clinic
Boyle 2023	Other	CRC[1]	NC	NC[5]	NC[5]	NC[5]	NC[5]	Malignant	NC[5]	NC[5]	Emergency/non-Elect.	NC[5]	NC[5]	NC[5]	NC[5]	Incompl. data	NC[5]	Outpatient clinic
Braga 1996	Other	CRC[1] and Upper GI[2]	18-75 yrs.	NC[5]	NC[5]	Curat.	NC[5]	Malignant	NC[5]	Limiting physical function	Emergency and palliative	NC[5]	NC[5]	NC[5]	Immune suppressive	NC[5]	Other	NR[12]
Braga 1999	Complication	CRC[1] and Upper GI[2] and HPB[3]	18-75 yrs.	NC[5]	NC[5]	Curat. and Elect.	NC[5]	Benign and malignant	NC[5]	Limiting physical function	Emergency/non-Elect.	Metastatic disease	NC[5]	NC[5]	Protocol violation	NC[5]	NC[5]	Outpatient clinic
Brunet 2017	QoL[13]	CRC[1]	≥ 18 yrs.	NC[5]	NC[5]	Curat.	Yes	Malignant	NC[5]	Unable to perform CPET[7]	NC[5]	Not resectable	Unable to exercise suff.	NC[5]	Neoadjuvant treatment	NC[5]	Unable to provide consent	NR[12]
Brunet 2021	Feasibility	Rectum tumor	Other	Other	NC[5]	NC[5]	Yes	Malignant	Distance from hospital	Limiting physical function	NC[5]	NC[5]	Unable to exercise suff.	NC[5]	NC[5]	NC[5]	Unable to provide consent and other	Outpatient clinic
Bruns 2019	Feasibility	CRC[1]	≥ 70 yrs.	Frailty Scale	NC[5]	Elect.	NC[5]	Malignant	NC[5]	Physical and psychiatric disease	Emergency/non-Elect.	NC[5]	NC[5]	NC[5]	NC[5]	NC[5]	Other	NR[12]
Burden 2011	Complication	CRC[1]	≥ 18 yrs.	NC[5]	NC[5]	Elect.	NC[5]	Malignant	NC[5]	Pregnancy	NC[5]	Not resectable	NC[5]	NC[5]	NC[5]	NC[5]	Unable to provide consent and other	Outpatient clinic
Burden 2017	Complication	CRC[1]	≥ 18 yrs.	NC[5]	No weight loss	Curat. and Elect.	NC[5]	Malignant	NC[5]	Diabetes and limiting physical or psychiatric function	NC[5]	NC[5]	NC[5]	NC[5]	Already consuming supplement	NC[5]	NC[5]	Outpatient clinic
Carli 2010	Physical improvement	CRC[1]	≥ 18 yrs.	NC[5]	NC[5]	Elect.	NC[5]	Benign and malignant	NC[5]	ASA[9]>III	NC[5]	NC[5]	Unable to exercise suff.	NC[5]	NC[5]	NC[5]	NC[5]	Outpatient clinic
Carli 2020	Complication	CRC[1]	≥ 65 yrs.	NC[5]	NC[5]	Curat.	NC[5]	Malignant	NC[5]	Limiting physical function	NC[5]	Metastatic disease	Unable to exercise suff.	NC[5]	NC[5]	NC[5]	Poor language skills	Outpatient clinic
De Klerk 2021	Complication	CRC[1]	≥ 65 yrs.	ASA[9] ≥ III	NC[5]	Elect.	NC[5]	NC[5]	Other	NC[5]	Emergency/non-Elect.	NC[5]	NC[5]	NC[5]	NC[5]	NC[5]	Other	Outpatient clinic

Englesbe 2017	Other	Abdomen surg. and Other	NC[5]	NC[5]	NC[5]	NC[5]	NC[5]	NC[5]	NC[5]	Limiting physical function	NC[5]	NC[5]	NC[5]	NC[5]	NC[5]	NC[5]	Other	Outpatient clinic
Estrada 2023	Other	CRC[1]	≥ 18 yrs.	NC[5]	NC[5]	NC[5]	NC[5]	Malignant	NC[5]	NC[5]	Additional resections	NC[5]	NC[5]	NC[5]	NC[5]	NC[5]	Other	Outpatient clinic
Franssen 2022	Feasibility	CRC[1]	≥ 18 yrs.	VAT[6] /V02 peak / CPET[7] criteria	NC[5]	NC[5]	NC[5]	Malignant	Mobile phone skills.	NC[5]	NC[5]	NC[5]	NC[5]	NC[5]	NC[5]	NC[5]	NC[5]	Phone
Furyk 2021	Physical improvement	CRC[1]	≥ 50 yrs.	Able to exercise and Other	NC[5]	NC[5]	NC[5]	Malignant	NC[5]	Psychiatric disorder/mental disorder/intellectual impairment	Emergency/non-Elect.	NC[5]	Unable to exercise suff.	NC[5]	NC[5]	NC[5]	Unable to provide consent	Outpatient clinic
Gillis 2014	Other	CRC[1]	≥ 18 yrs.	NC[5]	NC[5]	Curat.	NC[5]	Malignant	NC[5]	Limiting physical function	NC[5]	NC[5]	NC[5]	NC[5]	NC[5]	NC[5]	Poor language skills	Outpatient clinic
Gillis 2016	Physical improvement	CRC[1]	≥ 18 yrs.	NC[5]	NC[5]	Curat. and Elect.	NC[5]	Malignant	NC[5]	NC[5]	NC[5]	NC[5]	Unable to exercise suff.	Other	NC[5]	NC[5]	Poor language skills	Outpatient clinic
Gillis 2021	Other	CRC[1]	≥ 18 yrs.	NC[5]	NC[5]	NC	NC[5]	NC[5]	Computer skills	NC[5]	NC[5]	NC[5]	NC[5]	NC[5]	NC[5]	NC[5]	NC[5]	Outpatient clinic
Gonella 2024	Complication	CRC[1]	≥ 65 yrs.	Other	NC[5]	Elect.	NC[5]	Malignant	NC[5]	Physical and psychiatric disease	Emergency/non-Elect.	NC[5]	NC[5]	NC[5]	NC[5]	NC[5]	NC[5]	Other: via multidisciplinary meeting
Hara 2021	Complication	GIC[4]	NC[5]	NC[5]	NC[5]	Elect.	NC[5]	Malignant	NC[5]	Limiting physical function	Emergency/non-Elect.	Non-malignant	Unable to exercise suff.	NC[5]	NC[5]	NC[5]	NC[5]	Outpatient clinic
Hassan 2022	Other	CRC[1] and Upper GI[2] and HPB[3] and Lung and Other	≥ 70 yrs.	NC[5]	NC[5]	Elect.	NC[5]	NC[5]	NC[5]	Physical and psychiatric disease	NC[5]	NC[5]	NC[5]	NC[5]	NC[5]	NC[5]	NC[5]	Outpatient clinic
Heil 2023	Complication	CRC[1]	≥ 65 yrs.	ASA[9] ≥ III	NC[5]	Elect.	NC[5]	Malignant	NC[5]	Physical and psychiatric disease	Other	Metastatic disease	Unable to exercise suff.	NC[5]	Neoadjuvant treatment	NC[5]	Unable to provide consent and other	No informed consent retrieved
Heldens 2016	Feasibility	Rectum tumor	≥ 18 yrs.	NC[5]	NC[5]	Curat.	Yes	Malignant	NC[5]	Limiting physical function	NC[5]	NC[5]	Unable to exercise suff.	NC[5]	NC[5]	NC[5]	NC[5]	Outpatient clinic
Hernon 2021	Feasibility and Compliance	CRC[1]	≥ 18 yrs.	ASA[9] I-III	NC[5]	Curat. and Elect.	NC[5]	Malignant	NC[5]	Physical and psychiatric disease	NC[5]	NC[5]	NC[5]	NC[5]	NC[5]	NC[5]	NC[5]	Outpatient clinic
Huang 2016	Physical improvement	Other	NC[5]	NC[5]	NC[5]	NC[5]	NC[5]	Malignant	NC[5]	NC[5]	Other	NC[5]	NC[5]	NC[5]	NC[5]	Incompl. data	Other	Retrospective study
Ip 2024	Other	CRC and Upper GI[2]	Other	NC[5]	NC[5]	Elect.	NC[5]	NC	NC[5]	NC[5]	Other	Metastatic disease	Unable to exercise suff.	NC[5]	NC[5]	NC[5]	Other	Letter of invitation
Janssen 2020	Other	CRC and Other	≥ 70 yrs.	NC[5]	NC[5]	Elect.	NC[5]	Malignant	NC[5]	Limiting physical function	Emergency/non-Elect.	NC[5]	NC[5]	NC[5]	NC[5]	NC[5]	Other	Outpatient clinic
Karlsson 2019	Feasibility	CRC[1]	≥ 70 yrs.	NC[5]	NC[5]	Elect.	NC[5]	Malignant	NC[5]	Limiting physical function	Emergency/non-Elect.	NC[5]	NC[5]	NC[5]	NC[5]	NC[5]	Living distance	Outpatient clinic
Karlsson 2020	Other	CRC[1]	≥ 70 yrs.	NC[5]	NC[5]	NC[5]	NC[5]	Malignant	NC[5]	NC[5]	NC[5]	NC[5]	NC[5]	NC[5]	NC[5]	NC[5]	NC[5]	Outpatient clinic and phone
Kim 2009	Physical improvement	CRC[1]	≥ 18 yrs.	NC[5]	NC[5]	NC[5]	NC[5]	Benign and malignant	NC[5]	AD[11] limited/dependent	NC[5]	NC[5]	Unable to exercise suff.	NC[5]	Neoadjuvant treatment	NC[5]	NC[5]	Outpatient clinic
Koh 2020	Complication	Colon tumor	≥ 70 yrs.	Able to exercise	NC[5]	Curat.	NC[5]	Malignant	Other	NC[5]	Emergency/non-Elect.	NC[5]	NC[5]	NC[5]	NC[5]	NC[5]	NC[5]	Outpatient clinic
Kwok 2023	Physical improvement	GIC and Upper	NC[5]	NC[5]	NC[5]	Elect.	NC[5]	Malignant	NC[5]	NC[5]	NC[5]	NC[5]	NC[5]	NC[5]	NC[5]	Incompl. data	NC[5]	Outpatient clinic

		GI[2] and HPB[3] and Uro																
Lafaro 2020	Feasibility	GIC and Lung	≥ 65 yrs.	NC[5]	NC[5]	NC[5]	NC[5]	Malignant	Other	NC[5]	NC[5]	NC[5]	NC[5]	NC[5]	NC[5]	NC[5]	NC[5]	Outpatient clinic
Lee 2022	Complication	CRC[1]	≥ 18 yrs.	NC[5]	NC[5]	NC[5]	NC[5]	Malignant	Computer skills	NC[5]	NC[5]	NC[5]	NC[5]	NC[5]	NC[5]	NC[5]	NC[5]	NC[5]
Li 2013	Physical improvement	CRC[1]	≥ 18 yrs.	NC[5]	NC[5]	Elect.	NC[5]	Malignant	NC[5]	Limiting physical function	NC[5]	Metastatic disease	NC[5]	NC[5]	NC[5]	NC[5]	Poor language skills	Outpatient clinic
Lorca 2023	Physical improvement and feasibility	CRC[1]	NC[5]	NC[5]	NC[5]	NC[5]	NC[5]	Malignant	Mobile phone skills	NC[5]	NC[5]	NC[5]	NC[5]	NC[5]	NC[5]	Incompl. data	Other	Digital face-to-face session
Loughney 2019	Physical improvement and QoL[13] and Compliance	CRC[1] and Uro	≥ 18 yrs.	NC[5]	NC[5]	NC[5]	NC[5]	Malignant	NC[5]	Physical and psychiatric disease	NC[5]	NC[5]	Unable to exercise suff.	NC[5]	NC[5]	NC[5]	NC[5]	Outpatient clinic
Loughney 2021	Physical improvement	Rectum tumor	NC[5]	NC[5]	NC[5]	Elect.	Yes	Malignant	NC[5]	Limiting physical function	NC[5]	NC[5]	Unable to exercise suff.	NC[5]	NC[5]	NC[5]	Unable to provide consent and other	Outpatient clinic
MacFie 2000	Other	GIC[4]	NC[5]	NC[5]	NC[5]	Elect.	NC[5]	Benign and malignant	NC[5]	Physical and psychiatric disease	Emergency/non-Elect.	NC[5]	NC[5]	NC[5]	Neoadjuvant treatment	NC	Other	Outpatient clinic
Maňásek 2016	Complication	CRC[1]	≥ 18 yrs.	NC[5]	NC[5]	Curat.	NC[5]	Malignant	NC[5]	Limiting physical function	NC[5]	NC[5]	NC[5]	NC[5]	NC[5]	NC[5]	Other	Outpatient clinic
McIsaac 2022	Complication	Abdomen surg. and Other	Other	Frailty Scale	NC[5]	NC[5]	NC[5]	NC[5]	NC[5]	NC[5]	Other	Not resectable	NC[5]	NC[5]	NC[5]	NC[5]	Other	Outpatient clinic
Minnella 2020	Physical improvement	CRC[1]	NC[5]	NC[5]	NC[5]	NC[5]	NC[5]	Malignant	NC[5]	Limiting physical function	NC[5]	Metastatic disease	NC[5]	NC[5]	NC[5]	NC[5]	Poor language skills and other	Outpatient clinic
MolenaarCJL 2023	Complication	CRC[1]	≥ 18 yrs.	NC[5]	NC[5]	Curat. and Elect.	NC[5]	Malignant	NC[5]	ASA[9]>III and Limiting physical or psychiatric function	Emergency/non-Elect.	NC[5]	NC[5]	NC[5]	NC[5]	NC[5]	Poor language skills and other	Outpatient clinic
Morielli 2016	Physical improvement and feasibility	Rectum tumor	Other	No limiting co-morb.	NC[5]	NC[5]	Yes	Malignant	NC[5]	NC[5]	NC[5]	NC[5]	NC[5]	NC[5]	NC[5]	NC[5]	NC[5]	Outpatient clinic and phone
Morielli 2021	Feasibility	Rectum tumor	≥ 18 yrs.	Able to exercise and Other	NC[5]	Elect.	Yes	Malignant	NC[5]	NC[5]	NC[5]	NC[5]	NC[5]	NC[5]	NC[5]	NC[5]	NC[5]	Outpatient clinic
Mouch 2020	Other	Other	NC[5]	NC[5]	NC[5]	NC[5]	NC[5]	NC[5]	Other	NC[5]	Other	NC[5]	NC[5]	NC[5]	NC[5]	NC[5]	Other	Via phone, via video visit or in person
Moug 2018	Feasibility	Rectum tumor	≥ 18 yrs.	NC[5]	NC[5]	Curat.	Yes	Malignant	NC[5]	NC[5]	NC[5]	Metastatic disease	Unable to exercise suff.	NC[5]	NC[5]	NC[5]	Unable to provide consent	Outpatient clinic
Moya 2016	Complication	CRC[1]	≥ 18 yrs.	NC[5]	Normo-nourished	Elect.	NC[5]	Malignant	NC[5]	ASA[9]>III and Limiting physical or psychiatric function and pregnancy	Emergency/non-Elect.	NC[5]	Unable to consume supplement and food allergy	NC[5]	NC[5]	NC[5]	Other	NR[12]

Moya 2016	Complication	CRC[1]	≥ 18 yrs.	NC[5]	Normo-nourished	NC[5]	NC[5]	Malignant	MI surgery	ASA[9]>III and Limiting physical or psychiatric function	Emergency/non-Elect.	Organ failure	Unable to consume supplement	NC[5]	Already consuming supplement	NC[5]	NC[5]	Outpatient clinic
Northgraves 2020	Feasibility	CRC[1]	≥ 18 yrs.	NC[5]	NC[5]	Elect.	NC[5]	Benign and malignant	NC[5]	Limiting physical function	NC[5]	NC[5]	Unable to exercise suff.	NC[5]	NC[5]	NC[5]	NC[5]	NR[12]
Onerup 2020	Other	CRC[1]	Other	NC[5]	NC[5]	Elect.	NC[5]	Malignant	NC[5]	NC[5]	Other	NC[5]	Unable to exercise suff.	NC[5]	NC[5]	NC[5]	Unable to provide consent	Outpatient clinic
Onerup 2022	Other	CRC[1]	Other	NC[5]	NC[5]	Elect.	NC[5]	Malignant	NC[5]	NC[5]	Other	Metastatic disease	Unable to exercise suff.	NC[5]	NC[5]	NC[5]	Unable to provide consent and other	Outpatient clinic
Onerup 2024	Other	CRC[1]	Other	NC[5]	NC[5]	Elect.	NC[5]	Malignant	NC[5]	NC[5]	Other	NC[5]	Unable to exercise suff.	NC[5]	NC[5]	NC[5]	Unable to provide consent	Outpatient clinic
Peng 2021	Other	CRC[1]	Other	ASA[9] I-III	Other	NC[5]	NC[5]	NC[5]	NC[5]	Physical and psychiatric disease	Additional resections	NC[5]	NC[5]	NC[5]	NC[5]	No follow-up	NC[5]	Outpatient clinic
Pesce 2024	Physical improvement	Colon tumor	≥ 18 yrs.	NC[5]	NC[5]	Elect.	NC[5]	Malignant	NC[5]	ASA[9]>III and Limiting physical function	NC[5]	Metastatic disease	NC[5]	NC[5]	Neoadjuvant treatment	NC[5]	NC[5]	NR[12]
Rampam 2022	Physical improvement	Other	Other	Other	NC[5]	Elect.	NC[5]	NC[5]	Other	ADL[11] limited/dependent	NC[5]	NC[5]	NC[5]	NC[5]	NC[5]	NC[5]	Poor language skills and other	Outpatient clinic or phone
Rinninella 2021	Physical improvement	CRC[1]	≥ 75 yrs.	NC[5]	NC[5]	NC[5]	NC[5]	NC[5]	NC[5]	NC[5]	NC[5]	NC[5]	NC[5]	NC[5]	NC[5]	NC[5]	NC[5]	NR[12]
Sabajo 2024	Complication	CRC[1]	NC[5]	NC[5]	NC[5]	Curat. and Elect.	NC[5]	Malignant	NC[5]	NC[5]	NC[5]	NC[5]	NC[5]	NC[5]	NC[5]	NC[5]	Other	NR[12]
Serrano 2022	Compliance	GIC[4]	≥ 18 yrs..	NC[5]	NC[5]	Curat. and Elect.	NC[5]	Malignant	NC[5]	Physical and psychiatric disease	Emergency/non-Elect.	NC[5]	Unable to consume supplement	NC[5]	Immune suppressive	NC[5]	NC[5]	Outpatient clinic
Shelton 2021	Other	CRC[1]	≥ 18 yrs.	NC[5]	NC[5]	Elect.	NC[5]	NC[5]	Computer skills	NC[5]	NC[5]	NC[5]	NC[5]	NC[5]	NC[5]	Incompl. data	NC[5]	Outpatient clinic
Sier 2022	Feasibility	CRC[1]	Other	Able to exercise	BMI < 35 kg/m2	NC[5]	NC[5]	NC[5]	Other	Physical and psychiatric disease	Palliative	NC[5]	Unable to exercise suff. and food allergy	NC[5]	Already consuming supplement	NC[5]	Other	Outpatient clinic
Singh 2017	Other	Rectum tumor	NC[5]	No limiting co-morb. and Other	NC[5]	Curat.	NC[5]	Malignant	NC[5]	NC[5]	NC[5]	NC[5]	NC[5]	NC[5]	NC[5]	NC[5]	NC[5]	Outpatient clinic
Singh 2018	Feasibility	Rectum tumor	NC[5]	No limiting co-morb. and Other	NC[5]	Curat.	NC[5]	Malignant	Other	NC[5]	NC[5]	NC[5]	NC[5]	NC[5]	NC[5]	NC[5]	NC[5]	Outpatient clinic
Sorensen 2014	Other	CRC[1]	NC[5]	NC[5]	NC[5]	Elect.	NC[5]	Malignant	NC[5]	Pregnancy and physical or psychiatric disease and other	Emergency/non-Elect.	NC[5]	Unable to consume supplement and food allergy	NC[5]	NC[5]	NC[5]	Poor language skills and other	Outpatient clinic
Souwer 2018	Other	CRC[1]	≥ 75 yrs.	NC[5]	NC[5]	NC[5]	NC[5]	Malignant	NC[5]	Limiting physical function	Emergency/non-Elect.	Metastatic disease	Unable to consume supplement and food allergy	NC[5]	NC[5]	NC[5]	Other	Outpatient clinic

Suen 2022	Physical improvement	CRC[1]	≥ 18 yrs.	Other	NC[5]	Curat. and Elect.	NC[5]	Malignant	NC[5]	NC[5]	NC[5]	NC[5]	NC[5]	NC[5]	NC[5]	NC[5]	NC[5]	Outpatient clinic
Sun 2020	Other	GIC[4] and Lung	≥ 65 yrs.	NC[5]	NC[5]	NC[5]	NC[5]	Malignant	NC[5]	NC[5]	NC[5]	NC[5]	NC[5]	NC[5]	NC[5]	NC[5]	NC[5]	Outpatient clinic
Talbot 2024	Other	CRC[1]	≥ 18 yrs.	Other	Other	Curat. and Elect.	NC[5]	Malignant	NC[5]	Diabetes and limiting comorbidity	Emergency/non-Elect.	NC[5]	Unable to exercises suff. and food allergy	NC[5]	NC[5]	NC[5]	NC[5]	Outpatient clinic
Ten Cate 2024	Physical improvement	CRC[1]	NC[5]	Able to exercise	NC[5]	Elect.	NC[5]	Malignant	NC[5]	Limiting physical function	NC[5]	NC[5]	Unable to exercise suff.	NC[5]	NC[5]	NC[5]	Other	Outpatient clinic
Tew 2020	QoL[13]	Other	NC[5]	NC[5]	NC[5]	Elect.	NC[5]	NC	NC[5]	NC[5]	NC[5]	NC[5]	NC[5]	NC[5]	NC[5]	NC[5]	NC[5]	Screening forms were sent (clinic patients)
Valkenet 2016	Feasibility	GIC and Upper GI[2] and HPB[3]	NC[5]	NC[5]	NC[5]	Curat.	Yes	Malignant	NC[5]	NC[5]	NC[5]	NC[5]	NC[5]	NC[5]	NC[5]	NC[5]	NC[5]	Outpatient clinic
Van Rooijen 2019	Feasibility	CRC[1]	≥ 18 yrs.	NC[5]	NC[5]	Elect.	No	Malignant	NC[5]	ASA[9]>III and Limiting physical function	NC[5]	Metastatic disease	NC[5]	NC[5]	NC[5]	NC[5]	Unable to provide consent	Outpatient clinic
Van der Hulst 2021	Compliance	CRC[1]	≥ 70 yrs.	NC[5]	NC[5]	NC[5]	NC[5]	Malignant	NC[5]	NC[5]	Emergency/non-Elect.	Metastatic disease	Unable to exercise suff.	NC[5]	NC[5]	NC[5]	Other	Outpatient clinic
Van Exter 2023	Other	CRC[1] and Upper GI[2]	Other	NC[5]	NC[5]	Elect.	NC[5]	Malignant	NC[5]	Diabetes and limiting physical or psychiatric function	NC[5]	NC[5]	Unable to exercise suff.	NC[5]	NC[5]	Incompl. data	Poor language skills	Outpatient clinic
Waller 2022	Physical improvement	Abdomen surg.	≥ 18 yrs.	NC[5]	NC[5]	NC[5]	NC[5]	Malignant	NC[5]	Limiting physical function	NC[5]	NC[5]	NC[5]	NC[5]	NC[5]	NC[5]	Other	NR[12]
Wang 2022	Other	CRC[1]	NC[5]	NC[5]	NC[5]	NC[5]	NC[5]	NC	NC[5]	NC[5]	NC[5]	NC[5]	NC[5]	NC[5]	NC[5]	NC[5]	Unable to provide consent and other	Letter of invitation
Waterland 2021	Other	GIC[4] and Uro	≥ 18 yrs.	NC[5]	NC[5]	NC[5]	NC[5]	Malignant	NC[5]	Psychiatric disorder/mental disorder/intellectual impairment	NC[5]	NC[5]	NC[5]	NC[5]	NC[5]	NC[5]	Poor language skills	Outpatient clinic
Waterland 2022	Feasibility	Abdomen surg.	≥ 18 yrs.	VAT[6] /V02 peak / CPET[7] criteria	NC[5]	Elect.	NC[5]	Malignant	NC[5]	Limiting physical function and pregnancy	Emergency/non-Elect.	NC[5]	Unable to exercise suff.	NC[5]	NC[5]	NC[5]	Other	Outpatient clinic
West 2015	Physical improvement	Rectum tumor	≥ 18 yrs.	WHO[8] < 2	NC[5]	Curat.	Yes	Malignant	NC[5]	Unable to perform CPET[6]	NC[5]	Not resectable	Unable to exercise suff.	NC[5]	Neoadjuvant treatment	NC[5]	Unable to provide consent	NR[12]
Wong 2024	Feasibility	Other	≥ 50 yrs.	Walking dist. and Other	NC[5]	Elect.	NC[5]	NC	NC[5]	Physical and psychiatric disease	NC[5]	NC[5]	NC[5]	NC[5]	NC[5]	NC[5]	NC[5]	Outpatient clinic
Wooten 2021	Physical improvement and feasibility	Abdomen surg.	NC[5]	NC[5]	NC[5]	NC[5]	NC[5]	Malignant	NC[5]	Limiting physical function and Other	NC[5]	NC[5]	Unable to consume supplement	NC[5]	Already consuming supplement	NC[5]	Unable to provide consent	Outpatient clinic
Wu 2021	Feasibility	Other	≥ 18 yrs.	NC[5]	NC[5]	NC[5]	NC[5]	Malignant	NC[5]	NC[5]	NC[5]	NC[5]	NC[5]	NC[5]	NC[5]	Incompl. data	NC[5]	Phone or videocall (clinic patients)
Yang 2024	Other	CRC[1]	Other	ASA[9] I-III	NC[5]	Elect.	NC[5]	Malignant	NC[5]	ASA[9]>III and Limiting physical or	Emergency/non-Elect.	NC[5]	Unable to exercise suff.	NC[5]	NC[5]	NC[5]	Unable to	Outpatient clinic

										psychiatric function and pregnancy							provide consent	
Yoshida 2021	Feasibility	Rectum tumor	NC[5]	NC[5]	NC[5]	Elect.	NC[5]	Malignant	NC[5]	NC[5]	Prolonged intubation	Bowel obstruction	Unable to consume supplement	NC[5]	NC[5]	NC[5]	NC[5]	Retrospective examination

[1]CRC, colorectal cancer; [2]GI, gastro-intestinal; [3]HPB, hepato-pancreato-biliary; [4]GIC, gastro-intestinal cancer; [5]NC, no criterium; [6]VAT, ventilatory anaerobic threshold; [7]CPET, cardio-pulmonary exercise test; [8]WHO, World Health Organization; [9]ASA, American Society of Anesthesiologists; [10] NCRT, neoadjuvant chemo-radiotherapy; [11]ADL, activities of daily life; [12] NR, not reported; [13]QoL, quality of life.
