# Supplementary material for: Participation and Adherence to Prehabilitation Programs for Colorectal Cancer
Source: Nutrients. 2025 May 25;17(11):1792. doi: 10.3390/nu17111792 (PMC12157972; doi:10.3390/nu17111792)
Supplement: Supplementary file 1 [file nutrients-17-01792-s001.zip › 20250421PACE_SR_Patient_Ch_Table S2.pdf]

Table S2. Patients' characteristics

**Author**	**Total participants (n)**	**Prehab cohort (n)**	**Control cohort (n)**	**Participation prehab (%)**	**Male prehab (n)**	**Age prehab**	SD	Median	IQR Lower	IQR Upper	**BMI prehab**	SD	**Colon tumor prehab (%)**	**CRC prehab (%)**	**Rectum tumor prehab (%)**	**Open surg. (%)**	**MI surg. (%)**
Atoui 2023	102	43	46	51.8	58.1	65.6	12.63	NA[2]	NA[2]	NA[2]	25.8	NR[1]	55.8	NA[2]	44.2	11.6	88.4
Berkel 2022	74	28	29	56.0	57.0	74.0	7.00	NA[2]	NA[2]	NA[2]	29.8	4.1	NR[1]	NA[2]	NR[1]	0	82.0
Boereboom 2019	24	24	0	34.3	72.2	67.0	8.00	NA[2]	NA[2]	NA[2]	28.7	NR[1]	50.0	NA[2]	50.0	NR[1]	NR[1]
Bojesen 2022	8	8	0	88.9	50.0	80.0	NR[1]	NA[2]	NA[2]	NA[2]	26.2	NR[1]	100.0	NA[2]	0.00	14.30	85.7
Bousquet-Dion 2018	80	41	39	51.0	81.0	74.0	NA[2]	74.0	NR[1]	NR[1]	27.5	NR[1]	68.0	NA[2]	32.0	NR[1]	84.0
Boyle 2023	110	61	49	96.8	58.0	63.0	NR[1]	NR[1]	NR[1]	NR[1]	NR[1]	NR[1]	NR[1]	NA[2]	NA[2]	NR[1]	NR[1]
Braga 1996	40	20	20	NR[1]	55.0	64.1	12.50	NR[1]	NR[1]	NR[1]	NR[1]	NR[1]	35.0	NA[2]	25.0	100	0
Braga 1999	206	85	86	NR[1]	58.8	60.9	11.90	NR[1]	NR[1]	NR[1]	NR[1]	NR[1]	31.8	NA[2]	23.5	NR[1]	NR[1]
Brunet 2017	35	24	11	NR[1]	64.0	64.0	NR[1]	NR[1]	NR[1]	NR[1]	27.4	5.1	NR[1]	NA[2]	NR[1]	NR[1]	NR[1]
Brunet 2021	10	0	0	0	0.0	NA[2]	NA[2]	NA[2]	NA[2]	NA[2]	NA[2]	NA[2]	0	NA[2]	0	0	0
Bruns 2019	14	14	0	87.5	36.0	79.0	NR[1]	79.0	74.0	86.0	25.0	NR[1]	NR[1]	NA[2]	NR[1]	0	100
Burden 2011	125	59	66	77.6	63.0	64.5	13.90	NR[1]	NR[1]	NR[1]	25.0	4.8	NR[1]	NA[2]	NR[1]	3.7	NR[1]
Burden 2017	101	55	46	58.4	64.0	70.5	11.66	NR[1]	NR[1]	NR[1]	25.9	4.6	64.0	NA[2]	36.0	33.0	67.0
Carli 2010	133	66	67	83.6	58.6	61.0	16.00	NR[1]	NR[1]	NR[1]	28.0	6.0	63.0	63.0	NR[1]	76.0	24.0
Carli 2020	120	60	60	NR[1]	52.7	78.0	NR[1]	78.0	72.0	82.0	24.9	NR[1]	67.3	NA[2]	32.7	23.6	76.4
De Klerk 2021	351	76	275	57.0	51.0	75.0	NR[1]	75.0	NR[1]	NR[1]	26.2	5.4	74.0	NA[2]	26.0	1.0	99.0
Englesbe 2017	826	641	182	NR[1]	50.0	61.7	NR[1]	NR[1]	NR[1]	NR[1]	30.4	NR[1]	NR[1]	NA[2]	NR[1]	NR[1]	10.4
Estrada 2023	210	46	160	NR[1]	63.0	67.3	15.35	NA[2]	NA[2]	NA[2]	25.0	NR[1]	67.4	NA[2]	32.6	17.4	82.6
Franssen 2022	11	11	0	81.0	54.5	74.0	NA[2]	74.0	68.0	78.0	29.1	NR[1]	73.0	NA[2]	27.0	9.0	81.8
Furyk 2021	5	2	3	55.6	50.0	NR[1]	NR[1]	NR[1]	NR[1]	NR[1]	NR[1]	NR[1]	0	NA[2]	0	0	0
Gillis 2014	89	45	44	84.0	55.0	65.7	13.60	NR[1]	NR[1]	NR[1]	26.9	4.6	63.0	NA[2]	37.0	3.0	97.0
Gillis 2016	48	24	24	84.2	59.0	67.6	11.50	NR[1]	NR[1]	NR[1]	26.6	5.0	50.0	NA[2]	50.0	18.0	82.0
Gillis 2021	20	20	0	76.9	NR[1]	62.0	13.00	NR[1]	NR[1]	NR[1]	NR[1]	NR[1]	NR[1]	45.0	NR[1]	NR[1]	NR[1]
Gonella 2024	174	42	130	NR[1]	52.8	80.0	NR[1]	80.0	74.0	82.0	26.8	NR[1]	75.0	NA[2]	25.0	5.6	94.4
Hara 2021	45	45	0	20.7	60.0	NR[1]	NR[1]	NR[1]	NR[1]	NR[1]	NR[1]	NR[1]	17.8	NA[2]	15.6	57.8	NR[1]
Hassan 2022	370	185	185	NR[1]	55.1	65.9	12.50	NR[1]	NR[1]	NR[1]	29.2	6.6	10.8	NA[2]	10.8	NR[1]	NR[1]
Heil 2023	251	123	128	71.5	46.3	75.0	NR[1]	75.0	71.0	80.0	NR[1]	NR[1]	91.9	NA[2]	8.1	0.8	99.2
Heldens 2016	13	13	0	65.0	89.0	64.4	10.90	NR[1]	NR[1]	NR[1]	28.4	4.6	0	NA[2]	100	33.0	67.0
Hernon 2021	200	137	63	50.0	67.9	NR[1]	NR[1]	NR[1]	NR[1]	NR[1]	27.8	NR[1]	NR[1]	NA[2]	NR[1]	11.7	80.3

Huang 2016	26	26	0	NR[1]	85.0	67.7	9.60	NR[1]	NR[1]	NR[1]	29.3	6.0	NR[1]	58.0	NR[1]	NR[1]	NR[1]
Ip 2024	28	28	0	96.9	48.0	51.6	14.20	NR[1]	NR[1]	NR[1]	25.0	3.8	NR[1]	NA[2]	NR[1]	NR[1]	NR[1]
Janssen 2020	627	267	360	88.6	65.0	77.0	NA[2]	77.0	73.0	81.0	NR[1]	NR[1]	69.5	NA[2]	22.5	12.0	88.3
Karlsson 2019	23	11	12	35.0	40.0	83.5	NA[2]	83.5	76.0	85.0	NR[1]	NR[1]	90.0	NA[2]	10.0	30.0	70.0
Karlsson 2020	17	0	17	NR[1]	52.9	75.0	NR[1]	NA[2]	NA[2]	NA[2]	NR[1]	NR[1]	58.8	NA[2]	0	NR[1]	0
Kim 2009	21	14	7	NR[1]	64.0	55.0	15.00	NA[2]	NA[2]	NA[2]	26.6	5.9	NR[1]	NR[1]	NR[1]	NR[1]	NR[1]
Koh 2020	81	58	23	NR[1]	56.9	78.5	NA[2]	78.5	70.0	93.0	NR[1]	NR[1]	74.1	NA[2]	35.9	36.2	62.1
Kwok 2023	182	182	0	86.3	59.3	70.4	10.64	NA[2]	NA[2]	NA[2]	NR[1]	NR[1]	NR[1]	65.9	NR[1]	NR[1]	NR[1]
Lafaro 2020	34	34	0	75.6	68.8	68.0	NA[2]	68.00	66.0	84.0	NR[1]	NR[1]	NR[1]	NA[2]	NR[1]	NR[1]	NR[1]
Lee 2022	337	205	132	76.7	47.0	55.7	15.80	NA[2]	NA[2]	NA[2]	26.3	6.0	NR[1]	45.4	NR[1]	39.3	60.7
Li 2013	87	42	45	90.2	54.0	67.4	11.00	NA[2]	NA[2]	NA[2]	27.5	4.0	NR[1]	NA[2]	NR[1]	19.0	81.0
Lorca 2023	57	57	0	99.1	50.9	68.8	12.30	NA[2]	NA[2]	NA[2]	26.1	4.4	68.7	NA[2]	33.30	NR[1]	NR[1]
Loughney 2019	32	32	0	NR[1]	76.5	60.5	NR[1]	NR[1]	NR[1]	NR[1]	29.5	NR[1]	NR[1]	100.0	NR[1]	NR[1]	NR[1]
Loughney 2021	33	17	16	48.0	82.0	64.0	14.00	NA[2]	NA[2]	NA[2]	NR[1]	NR[1]	NR[1]	NA[2]	100	NR[1]	NR[1]
MacFie 2000	100	48	52	NR[1]	54.2	NR[1]	NR[1]	NR[1]	NR[1]	NR[1]	NR[1]	NR[1]	NR[1]	87.5	NR[1]	100	0
Maňásek 2016	52	52	105	NR[1]	NR[1]	64.0	9.90	NA[2]	NA[2]	NA[2]	NR[1]	NR[1]	NR[1]	NA[2]	NR[1]	NR[1]	NR[1]
McIsaac 2022	204	94	88	67.1	39.4	74.0	7.00	NA[2]	NA[2]	NA[2]	NR[1]	NR[1]	NR[1]	38.3	NR[1]	47.9	52.1
Minnella 2020	42	42	0	57.5	61.9	67.0	NR[1]	NA[2]	NA[2]	NA[2]	27.3	NR[1]	73.8	NA[2]	26.20	NR[1]	NR[1]
Molenaar 2023	269	136	133	39.3	50.4	69.0	NA[2]	69.0	60.0	77.0	26.3	NR[1]	82.1	NA[2]	17.9	4.1	95.1
Morielli 2016	18	18	0	56.0	66.7	57.5	10.40	NA[2]	NA[2]	NA[2]	28.7	4.2	0	NA[2]	100	NR[1]	NR[1]
Morielli 2021	36	18	18	27.5	61.0	56.0	14.00	NA[2]	NA[2]	NA[2]	27.5	4.9	0	NA[2]	100	NR[1]	NR[1]
Mouch 2020	1569	523	1046	NR[1]	46.3	70.0	NA[2]	70.0	66.0	75.0	NR[1]	NR[1]	20.8	NA[2]	3.40	NR[1]	NR[1]
Moug 2018	48	24	24	62.0	75.0	65.2	11.40	NA[2]	NA[2]	NA[2]	NR[1]	NR[1]	0	NA[2]	100.00	65.0	18.0
Moya 2016	128	64	64	98.4	49.2	69.0	NR[1]	NA[2]	NA[2]	NA[2]	NR[1]	NR[1]	NR[1]	NA[2]	NR[1]	0	100
Moya 2016	264	132	132	NR[1]	50.8	70.0	NR[1]	NA[2]	NA[2]	NA[2]	27.0	7.4	NR[1]	NR[1]	NR[1]	0	75.4
Northgraves 2020	23	11	11	18.4	40.0	64.1	10.50	NA[2]	NA[2]	NA[2]	30.3	4.3	30.0	NA[2]	60.0	60.0	40.0
Onerup 2020	217	106	111	68.2	57.0	69.2	12.30	NA[2]	NA[2]	NA[2]	25.5	3.7	61.0	NA[2]	39.0	43.0	57.0
Onerup 2022	761	379	382	65.3	60.0	69.0	11.00	NA[2]	NA[2]	NA[2]	26.0	4.2	50.0	NA[2]	50.0	37.0	56.0
Onerup 2024	761	379	382	65.0	61.0	67.5	10.90	NA[2]	NA[2]	NA[2]	25.9	3.9	NR[1]	NA[2]	NR[1]	NR[1]	NR[1]
Peng 2021	213	109	104	98.2	59.6	63.0	2.80	NA[2]	NA[2]	NA[2]	22.3	2.3	NR[1]	99.0	NR[1]	0	100
Pesce 2024	75	37	38	51.4	58.3	68.0	8.70	NA[2]	NA[2]	NA[2]	27.9	5.5	100.0	NA[2]	0	2.8	97.2
Rampam 2022	104	54	50	55.6	47.0	68.0	9.00	NA[2]	NA[2]	NA[2]	NR[1]	NR[1]	NR[1]	35.0	NR[1]	NR[1]	NR[1]

Rinninella 2021	302	166	136	NA[2]	48.2	80.8	4.05	NA[2]	NA[2]	NA[2]	26.5	3.9	NR[1]	94.6	NR[1]	4.2	95.8
Sabajo 2024	586	196	390	NR[1]	49.5	71.0	NA[2]	71.0	63.0	78.0	26.0	NR[1]	67.9	NA[2]	32.0	NR[1]	NR[1]
Serrano 2022	71	36	35	49.3	61.0	65.0	NA[2]	65.0	59.0	72.0	30.0	NR[1]	NR[1]	19.0	NR	NR	NR
Shelton 2021	227	227	0	74.9	45.5	55.1	15.30	NA[2]	NA[2]	NA[2]	26.3	6.1	43.5	NA[2]	NR[1]	40.60	59.40
Sier 2022	9	9	0	30.0	55.6	73.0	NA[2]	73.0	70.0	76.0	26.9	NR[1]	NR[1]	NA[2]	NR[1]	NR[1]	NR[1]
Singh 2017	12	12	0	70.6	50.0	54.4	12.90	NA[2]	NA[2]	NA[2]	26.1	4.2	0	NA[2]	100	NR[1]	NR[1]
Singh 2018	10	10	0	66.7	70.0	54.6	14.10	NA[2]	NA[2]	NA[2]	26.4	3.8	0	NA[2]	100	NR[1]	NR[1]
Sorensen 2014	148	74	74	82.7	66.7	68.0	14.00	NA[2]	NA[2]	NA[2]	28.0	7.0	71.4	NA[2]	28.6	NR[1]	NR[1]
Souwer 2018	224	86	138	85.0	49.0	80.6	6.20	NA[2]	NA[2]	NA[2]	26.0	3.8	70.0	NA[2]	30.0	17.0	83.0
Suen 2022	26	26	0	71.0	54.6	72.5	NA[2]	72.5	69.0	79.0	27.7	5.3	NR[1]	NA[2]	NA[2]	NR[1]	NR[1]
Sun 2020	34	34	0	NR[1]	NR[1]	68.0	NR[1]	NR[1]	NR[1]	NR[1]	NR[1]	NR[1]	47.1	47.1	NR[1]	NR[1]	NR[1]
Talbot 2024	26	13	13	NR[1]	NR[1]	NR[1]	NR[1]	NR[1]	NR[1]	NR[1]	NR[1]	NR[1]	NR[1]	NA[2]	NR[1]	NR[1]	NR[1]
Ten Cate 2024	101	101	0	64.7	51.5	69.7	12.70	NA[2]	NA[2]	NA[2]	27.0	4.5	64.4	NA[2]	35.6	4.0	82.1
Tew 2020	75	75	0	47.0	69.3	NR[1]	NR[1]	NR[1]	NR[1]	NR[1]	29.4	NR[1]	4.00	NR[1]	NR[1]	NR[1]	0
Valkenet 2016	115	115	53	68.4	57.4	62.3	10.70	NA[2]	NA[2]	NA[2]	26.6	9.5	23.5	NA[2]	0	NR[1]	NR[1]
Van Rooijen 2019	50	20	30	NR[1]	50.0	75.0	NA[2]	75.0	62.0	89.0	26.0	NR[1]	90.0	NA[2]	10.0	5.0	95.0
Van der Hulst 2021	334	124	210	99.4	53.2	80.0	NA[2]	80.0	76.0	83.8	26.6	NR[1]	68.5	NA[2]	31.5	4.8	96.30
Van Exter 2023	139	139	0	NA[2]	70.3	66.0	NA[2]	66.0	NR[1]	NR[1]	26.0	NR[1]	NR[1]	NA[2]	NR[1]	NR[1]	NR[1]
Waller 2022	22	11	11	67.0	36.4	55.5	NR[1]	NA[2]	NA[2]	NA[2]	30.0	NR[1]	NR[1]	63.60	NR[1]	NR[1]	NR[1]
Wang 2022	19	0	19	NA[2]	NR[1]	NR[1]	NR[1]	NR[1]	NR[1]	NR[1]	NR[1]	NR[1]	NR[1]	NA[2]	NR[1]	NR[1]	NR[1]
Waterland 2021	103	103	0	83.1	46.6	61.0	NA[2]	61.0	51.5	70.0	NR[1]	NR[1]	NR[1]	NA[2]	NR[1]	NR[1]	NR[1]
Waterland 2022	50	50	0	61.0	52.0	71.0	NA[2]	71.0	63.0	77.0	NR[1]	NR[1]	NR[1]	64.00	NR[1]	NR[1]	NR[1]
West 2015	39	22	17	NR[1]	64.0	64.0	NR[1]	NA[2]	NA[2]	NA[2]	27.4	5.1	0	NA[2]	100.0	65.0	35.00
Wong 2024	54	54	0	75.0	65.4	71.9	6.8	NA[2]	NA[2]	NA[2]	24.1	4.1	4.1	15.00	NR[1]	NR[1]	NR[1]
Wooten 2021	29	29	0	69.0	41.7	64.9	9.8	NA[2]	NA[2]	NA[2]	27.6	6.0	25.0	NA[2]	4.0	NR[1]	NR[1]
Wu 2021	139	139	0	76.0	52.0	67.0	NA[2]	67.0	60.0	73.0	27.9	NR[1]	NR[1]	62.10	NR[1]	NR[1]	NR[1]
Yang 2024	101	52	49	96.2	68.0	60.0	10.0	NA[2]	NA[2]	NA[2]	22.7	2.4	32.0	NA[2]	68.0	0	100
Yoshida 2021	74	42	32	NA[2]	57.0	67.2	11.2	NA[2]	NA[2]	NA[2]	22.4	3.5	0	NA[2]	100.0	0	100

[1]NR, not reported; [2]NA, not applicable.
