# Supplementary material for: Participation and Adherence to Prehabilitation Programs for Colorectal Cancer
Source: Nutrients. 2025 May 25;17(11):1792. doi: 10.3390/nu17111792 (PMC12157972; doi:10.3390/nu17111792)
Supplement: Supplementary file 1 [file nutrients-17-01792-s001.zip › 2025419PACE_Supplementary S1_QualityAssessmentQualitativeData.pdf]

Supplementary S1

EPPI Centre quality assessment

The following questions were answered to assess the quality of the studies.

1. Is the context of the study adequately described?
2. Are the aims of the study clearly reported?
3. Is there an adequate description of the sample used in the study and how the sample was identified and recruited?
4. Is there an adequate description of the methods used in the study to collect data?
5. Is there an adequate description of the methods of data analysis?
6. Is the study replicable from this report?
7. Do the authors avoid selective reporting bias? (e.g., do they report on all variables they aimed to study as specified in their aims/research questions?)
8. Are there ethical concerns about the way the study was done?
9. Were students and/or parents appropriately involved in the design or conduct of the study?
10. Is there sufficient justification for why the study was done the way it was?
11. Was the choice of research design appropriate for addressing the research question(s) posed?
12. Have sufficient attempts been made to establish the reliability of data collection methods and tools?
13. Have sufficient attempts been made to establish the validity of data collection tools and methods?
14. Have sufficient attempts been made to establish the reliability of data analysis?
15. Have sufficient attempts been made to establish the validity of data analysis?
16. To what extent are the research design and methods employed able to rule out any other sources of error/bias which would lead to alternative explanations for the findings of the study?
17. How generalizable are the study results?
18. Weight of evidence - A: Taking account of all quality assessment issues, can the study findings be trusted in answering the study question(s)?
19. Have sufficient attempts been made to justify the conclusions drawn from the findings so that the conclusions are trustworthy?
20. In light of the above, do the reviewers differ from the authors over the findings or conclusions of the study?

Gillis 2021

1. Accurate description of context; however, information about the center enrolling patients was limited.
2. The study's aims were well described: how patients prepared for surgery, their views, towards prehabilitation and how prehabilitation should be delivered.
3. A clear description of the sample and recruitment. However, it remains unclear why patients declined to participate in the study.
4. Adequate description of methods as semi-structured interviews were conducted and the interview questions were provided.
5. The method for data analysis was clearly described.
6. The replicability of this study was considered to be limited at certain points. Interviews could be repeated as questions were provided, also global methods for data analysis were given, but no coding template was described.
7. Limited risk of reporting bias, all intended aims were described.
8. No ethical concerns.
9. Appropriate involvement since the researcher conducting the interviews was a healthcare provider familiar with the patient group. Data were also analyzed by a patient with surgery-related experience and training in patient engagement. Researchers had no prior relationships with any of the participants.
10. Clearly described why the study was conducted.
11. A clear description of the research design and analytic plan; however, the choice for inductive analysis was not entirely clear.
12. The type of transcription methodology was adequately described; however, it was not clear whether this was reviewed or checked by patients.
13. Data were considered to be reliable for the patient population as broad inclusion criteria were used, and a standardized and clear interview format was used.
14. Collected data appeared to be saturated, however the transcription method was not provided.
15. Data analysis was described in detail, also data were analyzed by two researchers.
16. Unclear whether transcripts were checked by patients, nonetheless the description and conduct of data analysis were clear.
17. Results are quite generalizable as patients undergoing different types of CRC surgery were included, also no specific prehabilitation program was evaluated. On the other hand, it remains unclear how many male and female participants were included.
18. Medium trustworthiness
19. NA
20. NA

Karlsson 2020

1. The context was clearly described.
2. A clear aim of the study.
3. The used sample was clear, and also, identification and recruitment were described.
4. The method used was adequately described (semi-structured interviews), also interview questions were provided.
5. The data analysis was clearly described.
6. High replicability of the study.
7. Low risk of reporting bias, as results of all intended aims were described.
8. No ethical concerns.
9. Unclear whether there was any involvement of researchers in participants' care.
10. The authors clearly described why the study was performed in this manner.
11. Adequate description of research design.

12. Sufficient attempts to establish the reliability of data, i.e., an interview guide was used, and adequate skills of researchers.
13. Multiple researchers were involved in data collection, but transcripts were not sent to participants.
14. The reliability of the data was established by a clear approach and description of the coding process.
15. Researchers established the validity of data analysis i.e., by performing analysis by multiple assessors and discussing findings with other experts.
16. Patients were interviewed before surgery, this might have affected their answers, they could have had the feeling to answer socially desirable, it already involves patients who are willing to participate in a prehabilitation program.
17. Limited generalizability of data, some aspects are specific for this prehabilitation program, but general attitudes towards preoperative physical activities and exercise.
18. High level of trustworthiness.
19. NA
20. NA

Sier 2024

1. Clear and adequate description of the study context.
2. The aim of the study was clearly described.
3. The used sample was clearly described, also identification and recruitment were mentioned in the article.
4. The methods used in the study to collect data were adequately described.
5. Data analysis was adequately described.
6. Relatively high replicability as a shortened version of the used interview guide and coding template was provided.
7. Limited risk of reporting bias as variables aimed to study were specified.
8. No ethical concerns.
9. The participants appeared to be appropriately involved in the design and conduct of the study, also the researchers had no relationship with the participants.
10. The authors provided an explanation of the study design and approach used.
11. Appropriate research design for addressing the research questions.
12. The researchers aimed to establish the reliability of data collection by posing open-ended questions followed by questions from the interview guide and by conducting the interviews by a researcher not involved in their care. Also, verbatim transcription was used.
13. To establish the validity of data collection multiple participants were interviewed and the timeframe between the interview and participation in the prehabilitation program varied. Also, participants were asked to check the accuracy of the transcripts. On the other hand, the number of participants was low and in case of a long interval between study and prehabilitation there might be recall bias.
14. To establish the reliability of data analysis a consistent approach was used, a code framework was created and used, also, two researchers independently coded the transcripts. Data appeared to be saturated.
15. Clear description of the data analysis.
16. As the interview questions and results were directed to the prehabilitation program there is a limited risk of bias which would lead to alternative explanations for the findings of the study.
17. The study results are limitedly generalizable as the population is relatively small, even though almost all available patients were included. Also, some questions were specific to the studied prehabilitation program, which differs from other prehabilitation programs.
18. Medium to high trustworthiness.
19. NA
20. NA

Sun 2020

1. Adequate context description.
2. A clear description of the aims.
3. A clear description of how the sample was identified and recruited.
4. Partly. Notes were analyzed from sessions with physical therapists and occupational therapists but what exactly had to be noted or was noted is not clearly described.
5. Data analysis was briefly described but the program used was not clear. Unclear how information was provided.
6. Not possible to replicate the study.
7. Some selective reporting bias is possible as notes were analyzed this may introduce bias to the study.
8. No ethical concerns.
9. Unclear how patients and researchers were involved and related to each other and the conduct of the study.
10. The study does not justify the way the study was carried out.
11. The study design was appropriate for the research question.
12. Unclear how the reliability of data were checked.
13. Unclear how the validity of data were established.
14. The method of transcription and methods used to analyze data were provided; however, no coding template was attached.
15. Only one investigator read and coded all data and categorized these. However, two other researchers conducted validation, and data were discussed with the research team, this could contribute to the validity of data analysis.
16. No inclusion of lived experiences was described in the manuscript, this could introduce bias by only reporting and analyzing statements/notes from therapists.
17. The generalizability of the population is relatively high as several different patient groups were included, and results represent visions about physical activity in general, not about one specific program. On the other hand, the study only involved older aged patients and knowledge about patient characteristics remains limited as the given information is limited.
18. Low trustworthiness.
19. NA
20. NA

Talbot 2024

1. Quite a general but clear description of the context of the study.
2. The aims were clearly stated.
3. Adequate description of identification of patients and recruitment
4. The description of methods used to collect data were adequate.
5. The manuscript involves an adequate description of the methods of data analysis.
6. The study is replicable, i.e., authors provided the interview guide.
7. The variables mentioned in the aim of the study were also specified in the manuscript.
8. No ethical concerns.
9. Appropriate involvement of participants.
10. The study justifies the decision for qualitative study and the approach used.
11. Appropriate research design
12. The researchers attempted to establish the reliability of data collection by posing a narrative opening question followed by questions from the interview guide. Two researchers performed the interviews, both were not involved in the participants' care. Also, verbatim transcription was used.

13. To establish the validity of data collection multiple participants were interviewed and the timeframe was short to avoid recall bias. On the other hand, an artificial intelligence tool was used to transcribe the interviews; moreover, one of the two researchers checked the transcripts and interviews were not checked by patients.
14. The authors provided a detailed description of the development of the method and approach of data analysis, other experts were involved in the process. On the other hand, coding was performed by one researcher.
15. The description of the data analysis was clear.
16. As a relatively high number of patients was included and questions were specific to the study the risk of error/bias leading to alternative explanations for the findings is low.
17. Limited generalizability as the study sample and prehabilitation program are rather specific.
18. Medium to high trustworthiness.
19. NA
20. NA

Wang 2022

1. Clear information about the study setting and context.
2. Clear aim; to describe experiences and explore preferences for multimodal prehabilitation and gain insights into patients' experiences and preferences regarding surgical prehabilitation to advise a patient-centered multimodal prehabilitation program.
3. Clear eligibility criteria of samples to be used, identification and by whom they were recruited, and how.
4. Adequate description of methods used to collect data as focus groups were used to collect data, and questions were provided. How questions were developed/decided was not specified.
5. Adequate description of data analysis with verbatim transcriptions and transcripts underwent multiple iterations of data review and analysis, codes were created and organized into categories. A codebook was also provided.
6. High replicability of the study as focus groups were described in detail and the method for analysis and codebook was provided.
7. Limited risk of selective reporting bias, all data about main aims were provided; however, limited information was provided regarding outcomes of general questions.
8. No ethical concerns.
9. Unclear how involved participants were involved apart from the study elements.
10. A clear description of why the study was performed in this manner.
11. The qualitative design was described, but the reason why the authors had chosen direct content analysis was not described.
12. Limited attempts to establish reliability of data: transcripts were transcribed verbatim, but not checked by the participants for accuracy.
13. The validity of data and methods was considered sufficient as data and methodology were checked and supervised by the study coordinator.
14. A clear approach was used for analysis, a codebook was created and each stage was reviewed.
15. The validity of the analysis was established, i.e., via data analysis by multiple reviewers.
16. Low risk of error/bias causing alternative explanations for the findings as quite specific questions were posed.
17. Limited generalizability as only twelve patients were included, for both benign and malignant surgery indications. On the other hand, results provide a general overview of visions toward prehabilitation instead of an evaluation of experiences with prehabilitation.
18. Medium to a high level of trustworthiness.
19. NA
20. NA
