# Supplementary material for: Participation and Adherence to Prehabilitation Programs for Colorectal Cancer
Source: Nutrients. 2025 May 25;17(11):1792. doi: 10.3390/nu17111792 (PMC12157972; doi:10.3390/nu17111792)
Supplement: Supplementary file 1 [file nutrients-17-01792-s001.zip › 2025421PACE_Table S3_ExcPrehab.pdf]

Table S3. Characteristics of exercise interventions and prehabilitation.

Author	Min. L of prehab (w)	Training freq/w	Duration (min)	Supervised	Home-based	Personalized	Training type	Moderate training	E/Teleprehab	Group	Exercise intervention Control	Method of measuring compliance exercise intervention
Atoui 2023	4	3	50	No	No	Yes	Aerobic/cardio + resistance	No	No	No	General advice on healthy lifestyle	Self-reported; Supervisor
Berkel 2022	3	3	60	Yes	NR[1]	Yes	Aerobic/cardio + resistance	No	No	No	ERAS[3]	Supervisor
Boereboom 2019	1.5	4	NR[1]	Yes	No	No	Aerobic/cardio	No	No	No	NA[2]	Supervisor
Bojesen 2022	4	3	1	Yes	NR[1]	Yes	Aerobic/cardio + resistance	No	No	No	NA[2]	Supervisor
Bousquet-Dion 2018	4	5	35	Both	Both	Yes	Aerobic/cardio + resistance	No	No	NR[1]	Multimodal rehabilitation	Self-reported; Supervisor
Boyle 2023	NR[1]	NR[1]	NR[1]	No	Yes	Yes	Aerobic/cardio	No	No	No	NA[2]	Self-reported
Brunet 2017	6	3	40	Yes	NR[1]	NR[1]	NR[1]	No	No	No	NA[2]	Supervisor
Brunet 2021	12	3	75	Yes	No	Yes	Aerobic/cardio + resistance	No	No	No	NA[2]	Self-reported; Supervisor
Bruns 2019	2.6	7	7	No	Yes	No	Resistance	No	No	No	NA[2]	Self-reported
Carli 2010	4	3	30	NR[1]	NR[1]	Yes	Aerobic/cardio	No	No	No	Advise to walk daily for 30 min and daily breathing exercise	Self-reported
Carli 2020	4	4	30	Both	Both	No	Aerobic/cardio + resistance	Yes	No	No	Multimodal rehabilitation	Self-reported; Supervisor
De Klerk 2021	4	3	NR[1]	Yes	Both	NR	Aerobic/cardio	Yes	No	No	NA[2]	Supervisor
Englesbe 2017	2	NR[1]	NR[1]	No	Yes	No	Aerobic/cardio	Yes	Yes, completely	No	No intervention	Self-reported
Estrada 2023	4	3	40	No	NR[1]	Yes	Aerobic/cardio	Yes	No	No	Rehabilitation	Self-reported; Supervisor
Franssen 2022	NR[1]	4	30	Yes	Yes	Yes	Aerobic/cardio	No	Yes, completely	No	NA[2]	Supervisor
Furyk 2021	4	3	60	Yes	No	Yes	Aerobic/cardio + resistance	No	No	No	Standard care	Supervisor
Gillis 2014	4	3	NR[1]	No	Yes	Yes	Aerobic/cardio + resistance	No	No	No	Rehabilitation with 3x/wk home-based unsupervised exercise	Self-reported
Gillis 2021	NR[1]	NR[1]	NR[1]	NR[1]	NR[1]	NR[1]	NR[1]	NR[1]	NR[1]	NR[1]	NR[1]	NR[1]
Gonella 2024	3	2	NR[1]	NR[1]	NR[1]	NR[1]	Aerobic/cardio	No	No	No	NR[1]	NR[1]
Hara 2021	NR[1]	3	50	No	Yes	No	Aerobic/cardio + resistance	No	No	No	NA[2]	Self-reported
Hassan 2022	4	5	30	Both	Both	Yes	Aerobic/cardio + resistance	Yes	No	No	NR[1]	Self-reported; Supervisor
Heil 2023	3	3	60	Yes	No	NR	Aerobic/cardio	Yes	No	No	Standard care	Self-reported; Supervisor
Heldens 2016	5.5	2	60	Yes	No	Yes	Aerobic/cardio + resistance	No	No	No	NA[2]	Supervisor
Hernon 2021	3	5	NR[1]	Yes	Both	No	Aerobic/cardio + resistance	Yes	No	No	Standard care	Self-reported; Supervisor

Huang 2016	NR[1]	5	45	Both	Both	Yes	Aerobic/cardio + resistance	No	No	No	NA[2]	Self-reported
Ip 2024	NR[1]	NR[1]	NR[1]	NR[1]	NR[1]	No	Other	No	No	No	NA[2]	Self-reported
Janssen 2019	NR[1]	NR[1]	30	No	Yes	No	Aerobic/cardio + resistance	No	No	No	NA[2]	Self-reported
Janssen 2020	5	NA[2]	NA[2]	NA[2]	NA[2]	NA[2]	NA[2]	No	NA[2]	NA[2]	NA[2]	NR[1]
Karlsson 2019	2	3	60	Yes	Yes	Yes	Aerobic/cardio + resistance	Yes	No	No	Recommendation 150 min/w moderate physical activity	Self-reported; Supervisor
Karlsson 2020	NR[1]	NA[2]	NA[2]	NA[2]	NA[2]	NA[2]	NA[2]	NA[2]	NA[2]	NA[2]	NA[2]	NA[2]
Kim 2009	4	7	30	No	NR	Yes	Aerobic/cardio	No	No	No	Instructions on surgery preparation	Self-reported
Koh 2020	2	NR[1]	NR[1]	No	Yes	No	Resistance	No	No	No	ERAS[3]	Supervisor
Kwok 2023	NR[1]	7	30	Yes	Yes	No	Aerobic/cardio + resistance	No	No	No	NA[2]	Self-reported; Supervisor
Lafaro 2020	1	NR[1]	NR[1]	NR[1]	Yes	Yes	Other	No	No	No	NA[2]	Self-reported; Supervisor
Lee 2022	NR[1]	NR[1]	NR[1]	No	Yes	No	Aerobic/cardio + resistance	No	Yes, partly	No	ERAS[3]	Self-reported
Li 2013	NR[1]	3	30	No	NR[1]	No	Aerobic/cardio + resistance	No	No	No	ERAS[3]	Self-reported
Lorca 2023	3	2	NR[1]	NR[1]	NR[1]	Yes	Other	No	No	No	NA[2]	Self-reported
Loughney 2019	NR[1]	5	60	No	NR[1]	Yes	Aerobic/cardio	No	No	No	NR[1]	Supervisor
Loughney 2021	NR[1]	3	40	Yes	No	NR[1]	Aerobic/cardio	No	No	No	Standard care	Supervisor
McIsaac 2022	3	3	60	No	Yes	No	Other	No	No	No	WHO[4] Global Recommendations for Physical Activity and pedometer	Self-reported; Supervisor
Minnella 2020	4	3	35	Yes	No	Yes	Aerobic/cardio + resistance	No	No	No	NA	Supervisor
Moleaar 2023	4	NR[1]	60	NR[1]	NR[1]	Yes	Aerobic/cardio + resistance	No	No	No	ERAS[3]	Supervisor
Morielli 2016	6	3	NR[1]	Yes	NR[1]	Yes	Aerobic/cardio	Yes	No	No	NA[2]	Self-reported; Supervisor
Morielli 2021	NR[1]	3	NR[1]	Yes	NR	No	Aerobic/cardio	Yes	No	No	Standard activity	Self-reported; Supervisor
Mouch 2020	2	NR[1]	NR[1]	No	Yes	No	Aerobic/cardio	Yes	Yes, partly	No	NR[1]	Self-reported
Moug 2018	13	NR[1]	NR[1]	No	Yes	Yes	Aerobic/cardio	Yes	No	No	Standard care	Self-reported; Supervisor
Northgraves 2020	2	3	60	Yes	No	Yes	Aerobic/cardio + resistance	No	No	No	Standard care	Supervisor
Onerup 2020	2	7	30	No	Yes	No	Other	Yes	No	No	ERAS[3]	Self-reported
Onerup 2022	2	NA[2]	NA[2]	NA[2]	NA[2]	NA[2]	NA[2]	NA[2]	NA[2]	NA[2]	NA[2]	Self-reported
Onerup 2024	6	7	30	NA[2]	Yes	Yes	Aerobic/cardio	NA	No	No	NA[2]	Self-reported
Peng 2021	NR[1]	14	NR[1]	No	Yes	No	Resistance	No	No	No	ERAS[3]	Self-reported; Supervisor
Pesce 2024	4	4	NR[1]	Yes	Yes	Yes	Aerobic/cardio + resistance	No	No	No	Daily walking 30 min, and 5-10 min of aerobic exercises	Supervisor

Rampam 2022	3	NR[1]	NR[1]	No	Yes	No	Aerobic/cardio	Yes	No	No	Advice about sufficient physical activity	Self-reported; Supervisor
Sabajo 2024	4	3	NR[1]	Yes	No	NR[1]	Aerobic/cardio + resistance	No	No	No	Standard care	Supervisor
Shelton 2021	3	NR[1]	NR[1]	No	Yes	No	Aerobic/cardio + resistance	No	Yes, partly	No	NA[2]	Self-reported; Supervisor
Sier 2022	4	3	75	NR[1]	NR[1]	NR[1]	Aerobic/cardio + resistance	No	No	No	NA[2]	Supervisor
Singh 2017	16	2	60	Yes	Both	Yes	Aerobic/cardio + resistance	No	No	No	NA[2]	Self-reported; Supervisor
Singh 2018	10	4	60	Both	Both	Yes	Aerobic/cardio + resistance	Yes	No	NR	NA[2]	Supervisor
Souwer 2018	4	2	45	Yes	Both	NR	Aerobic/cardio + resistance	No	No	No	ERAS[3]	Self-reported; Supervisor
Suen 2022	2	2	60	Yes	Both	Yes	Aerobic/cardio + resistance	Yes	No	No	NA[2]	Self-reported; Supervisor
Sun 2020	NR[1]	NR[1]	NR[1]	NR[1]	NR[1]	NR[1]	NR[1]	NR[1]	NR[1]	NR[1]	NR[1]	Supervisor
Ten Cate 2024	3	3	32	NR[1]	NR[1]	Yes	Aerobic/cardio + resistance	No	No	No	NA[2]	Supervisor
Tew 2020	6	2	NR[1]	Yes	Both	No	NR	No	No	No	NA[2]	Self-reported; Supervisor
Valkenet 2016	NR[1]	2	30	Yes	No	No	Aerobic/cardio + resistance	Yes	No	Yes	NA[2]	Supervisor
van Rooijen 2019	4	3	NR[1]	Yes	Both	NR[1]	Aerobic/cardio + resistance	Yes	No	No	ERAS[3]	Self-reported; Supervisor
Van der Hulst 2021	4	2	45	Yes	Both	No	NR	No	No	No	NR[1]	Supervisor
Van Exter 2023	3	3	NR[1]	Yes	Yes	No	Aerobic/cardio + resistance	Yes	No	No	NA[2]	Self-reported; Supervisor
Waller 2022	2	5	30	No	Yes	Yes	Aerobic/cardio + resistance	No	No	No	NR[1]	Self-reported; Supervisor
Wang 2022	NR[1]	NR[1]	NR[1]	NR[1]	NR[1]	NR[1]	NR[1]	NR[1]	NR[1]	NR[1]	NR[1]	NA[2]
Waterland 2021	NR[1]	NR[1]	NR[1]	NR[1]	NR[1]	NR[1]	NR[1]	NR[1]	NR[1]	NR[1]	NR[1]	NA[2]
Waterland 2022	6	NR[1]	NR[1]	No	Both	Yes	Aerobic/cardio	No	No	No	Advice about sufficient physical activity	Self-reported
West 2015	6	3	40	Yes	No	Yes	Aerobic/cardio	No	No	No	CPET[5] and steps count	Supervisor
Wong 2024	4	2	90	Yes	No	NR	Aerobic/cardio + resistance	No	No	No	NA[2]	Supervisor
Wooten 2021	4	6	45	Yes	Yes	No	NR	Yes	Yes, partly	No	NA[2]	Self-reported
Wu 2021	NR[1]	3	150	NR[1]	NR[1]	No	Aerobic/cardio + resistance	Yes	Yes, partly	No	NA[2]	NR[1]
Yang 2024	NR[1]	14	NR[1]	No	Yes	No	Resistance	No	No	No	ERAS[3]	Self-reported; Supervisor

[1]NR, not reported; [2]NA, not applicable; [3]ERAS, enhanced recovery after surgery; [4]WHO, World Health Organization; [5]CPET, cardio-pulmonary exercise test.
