# Supplementary material for: Participation and Adherence to Prehabilitation Programs for Colorectal Cancer
Source: Nutrients. 2025 May 25;17(11):1792. doi: 10.3390/nu17111792 (PMC12157972; doi:10.3390/nu17111792)
Supplement: Supplementary file 1 [file nutrients-17-01792-s001.zip › 2025421PACE_Table S4_NutrPrehab.pdf]

Table S4. Characteristics of nutritional interventions and prehabilitation

Author	Min. L of prehab (w)	Supplement	Protein intake	Personalized diet	Screening	Enteral feeding	Other	Type of nutritional intervention control cohort	Method of measuring compliance nutritional intervention
Atoui 2023	4	Preop protein supplement	Yes, ESPEN[4] guideline	Yes	No	No	No	NR[1]	Self-reported
Bojesen 2022	4	Other	No	No	No	No	No	NA[3]	NR[1]
Bousquet-Dion 2018	4	No	Other	No	Yes	No	No	NR[1]	Self-reported; Supervisor
Boyle 2023	NR[1]	No	No	Yes	Yes	No	No	NR[1]	Self-reported
Braga 1996	1	Preop + postop liquid ONS[2]	No	No	No	No	No	1 L /d liquid control diet for 7 consecutive days and postoperative control diet	Supervisor
Braga 1999	1	Preop + postop liquid ONS[2]	No	No	No	No	No	1x/day liquid diet pre- and postop with placebo.	NR[1]
Bruns 2019	2.6	Other	Yes, ESPEN[4] guideline	No	No	No	No	NA[3]	Self-reported
Burden 2011	1.4	Preop liquid ONS[2]	No	No	No	No	No	Information on healthy nutrition	Self-reported
Burden 2017	1	Preop liquid ONS[2]	No	No	No	No	No	Placebo oral supplementation	Self-reported
Carli 2020	4	Other	Other	NA[3]	No	No	No	Similar program for rehabilitation	Self-reported
De Klerk 2021	4	No	Other	No	No	No	No	NA[3]	NR[1]
Englesbe 2017	2	No	No	No, general information	No	No	No	NR[1]	Self-reported
Estrada 2023	4	No	No	Yes	Yes	No	No	Rehabilitation	NR[1]
Franssen 2022	NR[1]	No	Yes, ESPEN[4] guideline	No	Yes	No	No	NA[3]	Self-reported
Furyk 2021	4	No	No	No, general information	No	No	No	NR[1]	NR[1]
Gillis 2014	4	Preop protein supplement	Yes, ESPEN[4] guideline	Yes	No	No	No	Whey protein postop	NR[1]
Gillis 2016	4	Preop + postop protein supplement	Yes, ESPEN[4] guideline	No	No	No	No	1x/day placebo supplement 4 weeks before and after surgery	Self-reported
Gillis 2021	NR[1]	NR[1]	NR[1]	NR[1]	NR[1]	NR[1]	NR[1]	NA[3]	NA[3]
Gonella 2024	3	Other	No	No	No	No	No	NR[1]	NR[1]

Hara 2021	NR[1]	No	Yes, ESPEN[4] guideline	No	No	No	No	NA[3]	NR[1]
Ip 2024	NR[1]	No	No	No, general information	No	No	No	NA[3]	Self-reported
Janssen 2020	5	NA[3]	NA[3]	NA[3]	NA[3]	NA[3]	NA[3]	NR[1]	NR[1]
Koh 2020	2	Other	No	No	No	No	No	ERAS[5]	Supervisor
Kwok 2023	NR[1]	No	Yes, ESPEN[4] guideline	No	No	No	Yes	NA[3]	NR[1]
Lee 2022	NR[1]	No	No	No, general information	No	No	No	ERAS[5]	Self-reported
Li 2013	NR[1]	Preop protein supplement	Other	No	No	No	No	ERAS[5]	Self-reported
MacFie 2000	1	Preop liquid ONS[2]	No	No	No	No	No	Postop oral supplement of at least 2x200ml for 7 days or no supplement	Self-reported
Maňásek 2016	1.4	Preop + postop liquid ONS[2]	No	No	No	No	No	Standard care	NR[1]
McIsaac 2022	3	No	No	No, general information	No	No	No	Information on healthy nutrition	NR[1]
Minnella 2020	4	Preop protein supplement	Other	Yes	No	No	No	NA[3]	Self-reported
Mole888arCJL 2023	4	Preop protein supplement	Other	No	No	No	No	ERAS[5]	Self-reported
Mouch 2020	2	No	No	No, general information	No	No	No	NR[1]	NR[1]
Moya 2016	1	Preop + postop liquid ONS[2]	No	No	No	No	No	Information on healthy nutrition	Self-reported
Moya 2016	1	Preop + postop liquid ONS[2]	No	No	No	No	No	7 days 2x400ml with hypercaloric, high-protein supplement (HHS)-SUPRESSI of Vegenat	Self-reported
Rinninella 2021	3	No	No	Yes	No	No	No	NA[3]	Self-reported
Sabajo 2024	4	Other	No	No	No	No	No	NR[1]	NR[1]
Serrano 2022	NR[1]	Other	No	No	No	No	NA	Placebo oral supplement	Supervisor
Shelton 2021	3	No	No	No, general information	No	No	No	NA[3]	Self-reported
Sier 2022	4	Other	No	No	No	No	Yes	NA[3]	Self-reported; Supervisor
Sorensen 2014	1	Other	No	No	No	No	No	ONS[2] for 7 days pre-op	NR[1]
Souwer 2018	4	No	Yes, ESPEN[4] guideline	Yes	Yes	No	No	ERAS[5]	NR[1]
Suen 2022	2	No	No	Yes	No	No	No	Information on healthy nutrition	Self-reported

Talbot 2024	NR[1]	Preop liquid ONS[2]	NA	NA	NA	NA	No	Standard care	Self-reported
Ten Cate 2024	3	Preop protein supplement	Yes, ESPEN[4] guideline	No	No	No	No	NA[3]	NR[1]
Tew 2020	6	Other	No	No	Yes	No	No	NA[3]	NR[1]
van Rooijen 2019	4	Preop protein supplement	Yes, ESPEN[4] guideline	No	No	No	No	ERAS[5]	Self-reported
Van Exter 2023	3	Preop protein supplement	Yes, ESPEN[4] guideline	No	Yes	No	No	NA[3]	Self-reported; Supervisor
Waller 2022	2	No	No	No, general information	No	No	No	NR[1]	Self-reported
Wang 2022	NR[1]	NR[1]	NR[1]	NR[1]	NR[1]	NR[1]	NR[1]	NR[1]	NA[3]
Waterland 2021	NR[1]	NA[3]	NA[3]	NA[3]	NA[3]	NA[3]	NA[3]	NA[3]	NA[3]
Wong 2024	4	No	Yes, ESPEN[4] guideline	Yes	Yes	No	No	NR[1]	NR[1]
Wooten 2021	4	Other	No	No	No	No	No	NA[3]	Self-reported
Wu 2021	NR[1]	No	Yes, ESPEN[4] guideline	No, general information	No	No	No	NA[3]	NR[1]
Yoshida 2021	NR[1]	Preop liquid ONS[2]	No	No	No	No	No	NA[3]	Supervisor

[1]NR, not reported; [2]ONS, oral nutritional supplements; [3]NA, not applicable; [4]ESPEN, European Society of Parenteral and Enteral Nutrition; [5]ERAS, enhanced recovery after surgery.
